# Effects of Measurement Temperature on Radioluminescence Processes in Cerium-Activated Silica Glasses for Dosimetry Applications

**DOI:** 10.3390/s23104785

**Published:** 2023-05-16

**Authors:** Ismail Zghari, Hicham El Hamzaoui, Bruno Capoen, Franck Mady, Mourad Benabdesselam, Géraud Bouwmans, Damien Labat, Youcef Ouerdane, Adriana Morana, Sylvain Girard, Aziz Boukenter, Mohamed Bouazaoui

**Affiliations:** 1 Univ-Lille, CNRS, UMR 8523-PhLAM-Physique des Lasers Atomes et Molécules, F-59000 Lille, France; ismail.zghari@univ-lille.fr (I.Z.); hicham.el-hamzaoui@univ-lille.fr (H.E.H.); geraud.bouwmans@univ-lille.fr (G.B.); damien.labat@univ-lille.fr (D.L.); mohamed.bouazaoui@univ-lille.fr (M.B.); 2 UMR 7010, Institut de Physique de Nice (INPHYNI), Université Côte d’Azur, 06108 Nice, France; franck.mady@unice.fr (F.M.); mourad.benabdesselam@unice.fr (M.B.); 3 Laboratoire Hubert Curien UMR 5516, Institut d’Optique Graduate School, Université Jean Monnet Saint-Etienne, CNRS, F-42023 Saint-Etienne, France; ouerdane@univ-st-etienne.fr (Y.O.); adriana.morana@univ-st-etienne.fr (A.M.); sylvain.girard@univ-st-etienne.fr (S.G.); aziz.boukenter@univ-st-etienne.fr (A.B.)

**Keywords:** dosimetry, cerium, silica glass, optical fiber sensors, radioluminescence, modeling

## Abstract

Cerium-doped-silica glasses are widely used as ionizing radiation sensing materials. However, their response needs to be characterized as a function of measurement temperature for application in various environments, such as in vivo dosimetry, space and particle accelerators. In this paper, the temperature effect on the radioluminescence (RL) response of Cerium-doped glassy rods was investigated in the 193–353 K range under different X-ray dose rates. The doped silica rods were prepared using the sol-gel technique and spliced into an optical fiber to guide the RL signal to a detector. Then, the experimental RL levels and kinetics measurements during and after irradiation were compared with their simulation counterparts. This simulation is based on a standard system of coupled non-linear differential equations to describe the processes of electron-hole pairs generation, trapping-detrapping and recombination in order to shed light on the temperature effect on the RL signal dynamics and intensity.

## 1. Introduction

Optically activated silica glasses attracted huge interest as promising scintillating materials for radiation dosimetry in several fields of application ranging from medical physics (radiotherapy and diagnosis) to nuclear energy and astrophysics [1,2,3,4,5,6,7]. One of the advantages of such scintillators is that silica glasses, showing excellent chemical, thermal and mechanical properties, can be easily drawn into optical fibers. Thanks to their small size, intrinsic immunity to electromagnetic perturbations, flexibility and ability to be remotely interrogated, they allowed the development of point dosimeters with excellent spatial resolutions and with the possibility to operate in hazardous, narrow and constrained environments. Moreover, they enable good reproducibility, sensitivity to low dose rates, resistance to high dose levels irradiations and linear response in a wide dose rate range [3,5,6,8]. This is very important to ensure quality control of radiation facilities and the safety of workers around.

Ce^3+^-activated silica glasses are highly sensitive luminescent materials for ionizing radiation dosimetry [1,2,3]. Moreover, due to the ability of Ce ions to reduce the formation of radiation-induced color centers [9,10,11], cerium-doped silica glasses have been widely studied under ionizing radiation and are considered promising candidates for radiation dosimetry [1,2,3,5,6]. In this domain, the radioluminescence (RL) technique is of great interest. Indeed, the scintillation signal from the radiation-exposed probe can be recorded, allowing real-time dose-rate measurements. This should allow in vivo dosimetry, which consists of measuring directly on the patient the deposited radiation dose during radiotherapy treatment.

When a scintillating material is subjected to ionizing radiation, free electrons and holes are created. These electron-hole pairs could be transferred to the luminescence center, intrinsic centers or dopants such as rare-earth ions used as activators, which finally emit photons in ultraviolet, visible or near-infrared domains. Additionally, a proportion of the generated charge carriers are captured in the forbidden energy bandgap by several kinds of traps. Stable electron or hole traps are deep enough to remain filled, which means that no de-trapping occurs at the measurement temperature. Unstable traps are shallow enough to quickly release the trapped charges. Finally, metastable traps can be defined by their intermediate energy depth in the bandgap, allowing carriers to be detrapped after a certain delay.

In this context, several RL measurements have been carried out in cerium-doped silica glasses in different previous studies [12,13,14,15]. Recently, we have reported on the simulation of the RL signal as a function of the dose rate at room temperature (RT) [16]. However, a theoretical approach is still needed, in particular, to explain the kinetics and levels of the RL as a function of temperature. The present paper is devoted to the influence of the measurement temperature on the RL signal in the Ce-doped silica glass scintillator. To this end, RL spectral measurements on Ce^3+^-doped silica glass at different dose rates and temperatures (between 193 and 353 K) were carried out to identify the recombination center. Moreover, the RL signal was measured as a function of time in the same temperature conditions. These RL signal traces have been modeled, based on a set of rate equations, with the aim of reproducing experimental results.

## 2. Materials and Methods

### 2.1. Studied Sample

A sample of Ce^3+^-doped vitreous silica rods was prepared using the sol-gel method at FiberTech Lille platform of the University of Lille, as described elsewhere [3]. Then, this rod was drawn at a temperature of about 2000 °C down to a submillimeter diameter (about 220 μm). For RL measurements, the experimental setup was composed of 1 cm-long piece of the Ce^3+^-doped cane spliced to 5 m long and 220 µm diameter radiation-hard multimode optical fiber. This fiber, supplied by Exail, was used as a transport waveguide for the RL signal.

### 2.2. RL Measurements

The external X-ray beam was delivered by the LABHX facility of Laboratoire Hubert Curien, operating at 100 kV and generating photons of ~40 keV average energy fluence [17]. The X-ray dose rate was driven by the electric current of the equipment. The 1 cm-long piece of the doped cane was placed in a calibrated position of the X-irradiator. Under X-rays, the RL signal was then guided through the transport fiber toward a photomultiplier module (PMT, H9305-13 Hamamatsu). The acquisition is set with a numerical oscilloscope (Rohde & Schwarz RTM 3004). The dose rate at the various locations has been measured with an ionization chamber. All the values of dose rate and dose have been converted by considering the ratio between mass attenuation coefficients *μ*/*ρ* of water and silica, where *μ* and *ρ* are the photon attenuation coefficient and the medium density, respectively. This ratio has been estimated to be 2.5 for the used X-ray energies. Doses are consequently given in Gy(silica) unit.

**Figure 1:**
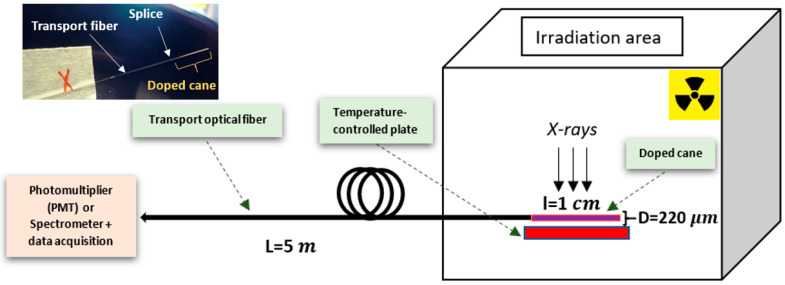
Illustration of the experimental setup used to characterize the RL response of the Ce^3+^-doped cane exposed to X-ray beam. The inset shows a photograph of the sample connected to the transport fiber.

RL spectra were collected, at different temperatures, after exchanging the PMT detector with a UV/VIS mini-spectrometer (Ocean Insight QE PRO) in the setup shown in Figure 1. For each RL spectrum, the dark signal, due to the device, was subtracted before analysis. The temperature of the sample was varied by sticking the transport fiber to a “temperature-controlled plate” associated with a thermocouple (type K with uncertainty of 1%), and for each temperature, before irradiation, a thermalization time of 15 min was adopted.

## 3. Results and Discussion

### 3.1. Experiment

#### RL Measurement

A. RL signal

After placing the sample on the temperature-controlled plate and connecting the transport fiber to the PMT, we performed the measurements in temperature ranges from 293 to 193 K, then from 313 to 353 K (295, 233, 193, 313, 333, 353 K). At each given temperature, the sample was exposed to four different dose rates (5, 50, 100, and 150 mGy/s). In this measurement part, the sample was exposed to X-rays for 120 s, and the data acquisition was continued for 60 s after the irradiation to allow the detection of the phosphorescence signal. Furthermore, in our measurement conditions, the measured RL signals were influenced neither by the Cerenkov effect since we work with an irradiator with an average energy fluence of 40 keV, nor by other stem effects coming from the transport fiber since it was protected by a thick lead cover.

**Figure 2:**
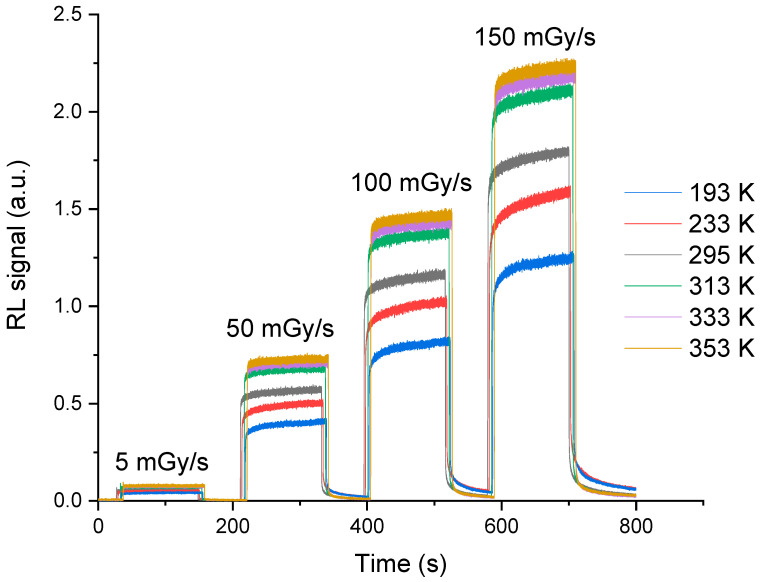
RL signal response of the cerium-doped silica cane spliced to a transport fiber for different X-ray dose rates and at temperature ranging from 193 K to 353 K.

Figure 2 shows the evolution of the RL signal as a function of time, recorded from the Ce-doped sample, for different dose rates and at different temperatures. Independently of the dose rate and temperature, at the beginning of the irradiation, the experimental RL signal response is characterized by an initial abrupt jump from the background level, then it undergoes a slower rise to finally tend toward a plateau (pseudo-plateau as the equilibrium is not yet reached). Moreover, immediately after stopping the irradiation, the signal shows a fast decrease, then exhibits an afterglow behavior (phosphorescence).

**Figure 3:**
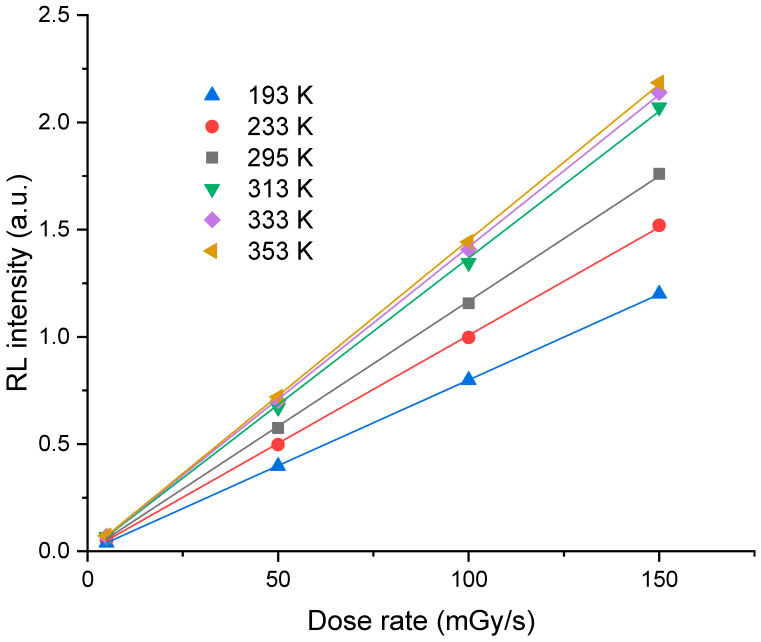
Dose rate dependence of the RL response of the cerium-doped silica cane exposed to irradiation at different X-ray dose rates obtained at different temperatures ranging from 193 K to 353 K. Each point represents the average RL signal in the pseudo-plateau region.

In Figure 3, we have plotted the evolution of the mean RL signal, in the pseudo-plateau region, as a function of the X-ray dose rate for different measurement temperatures. It shows a linear behavior as a function of the dose rate for all the measurement temperatures. Furthermore, it can be seen from Figure 2 and Figure 3 that the RL response increases with temperature.

**Figure 4:**
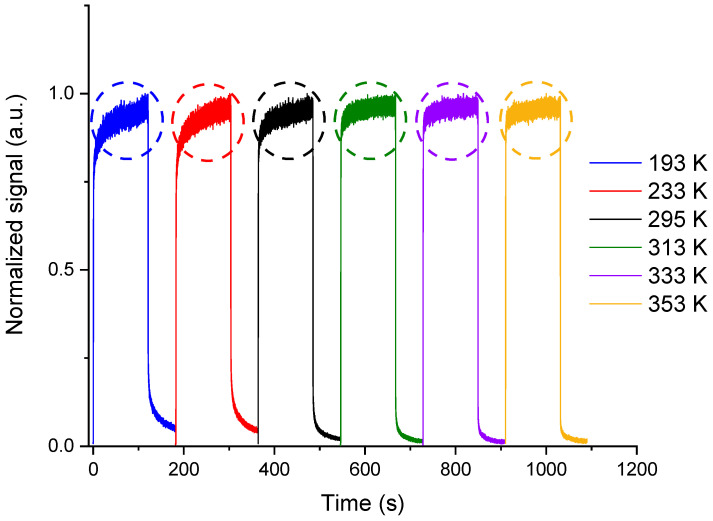
Example of normalized RL signal dynamics recorded from the cerium-doped silica sample at a dose rate of 50 mGy/s at different measurement temperatures. The dotted circles show the pseudo-plateau zones.

**Figure 5:**
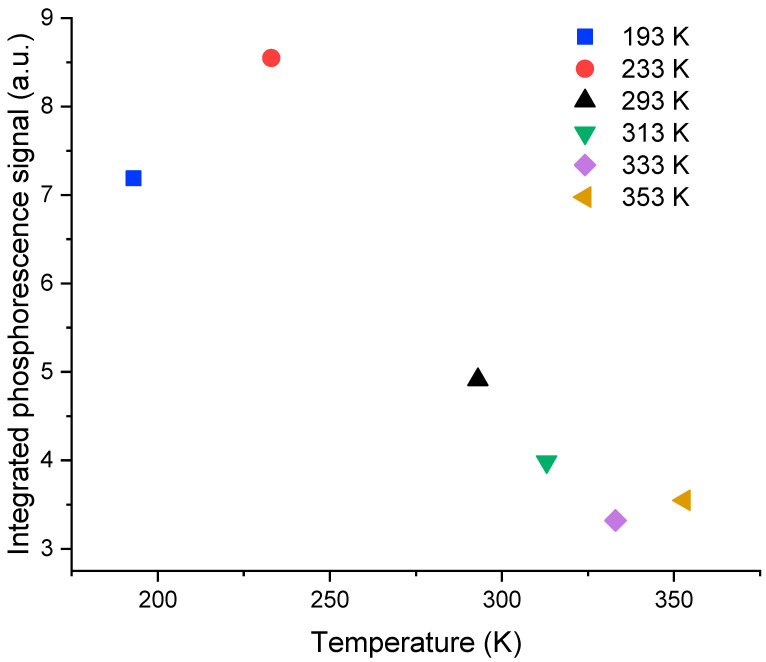
Integrated phosphorescence signal response of the cerium-doped silica cane, 60 s after the end of irradiation, as a function of the temperature ranging from 193 K to 353 K at a dose rate of 50 mGy/s.

Figure 4 illustrates the shape of the normalized RL signals for a dose rate of 50 mGy/s at the various measurement temperatures. Note that the shape changes from one temperature to another in the pseudo-plateau regime limited by the dotted circles. As already explained in [16] for RL measurements at room temperature, the signal dynamics is related to the trapping-detrapping of electrons by unstable traps in each measurement condition (time, temperature, etc.). For example, in this pseudo-plateau area, below room temperature, the slope of the signal rise is larger than the ones observed above room temperature. Figure 5 presents the integral of the signal over 60 s just after the end of the irradiation for a dose rate of 50 mGy/s at different temperatures. The phosphorescence at low temperatures is more intense than that at high temperatures. Note that these behaviors, which are the same for other dose rates, will be discussed and interpreted in Section 3.2 in light of simulation results.

B. RL spectra

In this measurement part, we kept the same experimental configuration as before; we just replaced the PMT with a UV-Visible spectrometer module. The sample was exposed with the same dose rates and at the same temperatures as before, keeping the same order of measurement temperatures.

**Figure 6:**
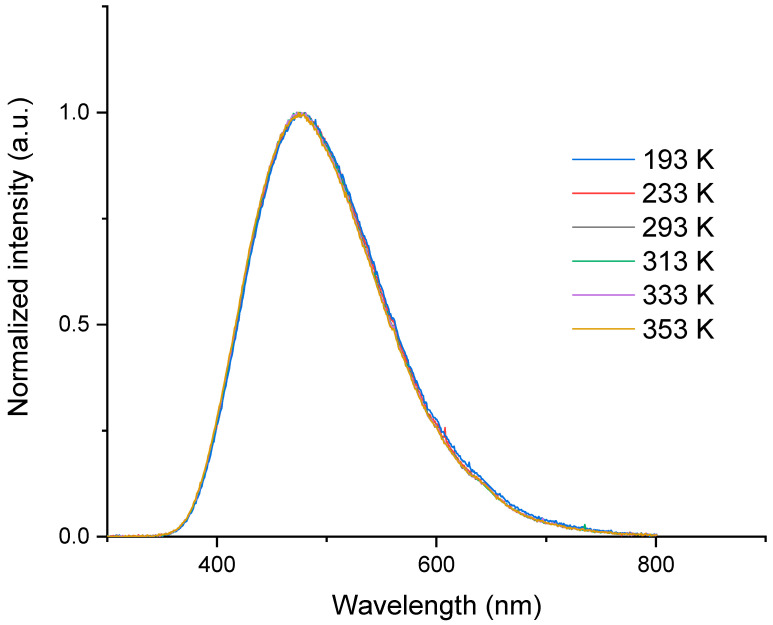
Normalized RL spectra of the cerium-doped silica cane spliced to a transport fiber obtained at different temperatures, ranging from 193 K to 353 K, under 50 mGy/s dose rate.

In Figure 6, we have plotted the normalized RL spectra at different measurement temperatures for a 50 mGy/s dose rate. It was observed that the spectra remain identical in this interval of temperatures. This was also obtained for the other three investigated dose rates. The broad band centered around 460 nm is attributed to the allowed transitions 5d → 4f of Ce^3+^ ions. It should be stressed that the band maximum follows a linear behavior as a function of the dose rate at each temperature. Furthermore, the evolution of this maximum with temperature is identical to that of the RL signal measured with a PMT, as illustrated in Figure 3.

These results show that the Ce^3+^ ions constitute the recombination centers (RC) at the origin of the RL signal during irradiation. Moreover, the independence of the spectral shape to dose rate and temperature indicates that the local silica environment around the Ce^3+^ ions did not change. It can also be concluded, given the low dose accumulated in the sample and the unchanged form of the spectra, that there is no discernable impact of the radiation-induced attenuation effect (RIA) in this spectral range from 350 to 700 nm. This is the reason why RIA has not been considered in the model of this glass described in Section 3.2.

### 3.2. Model and Simulation

The kinetics model used in this section to simulate our RL experimental results is based on the previously published one reported in [16] for measurements performed at RT. The difference here relies on the introduction of additional traps in the model in order to be able to simulate the experimental results for different measurement temperatures and to follow the thermal evolution of the charge carriers’ populations in the trap levels.

**Figure 7:**
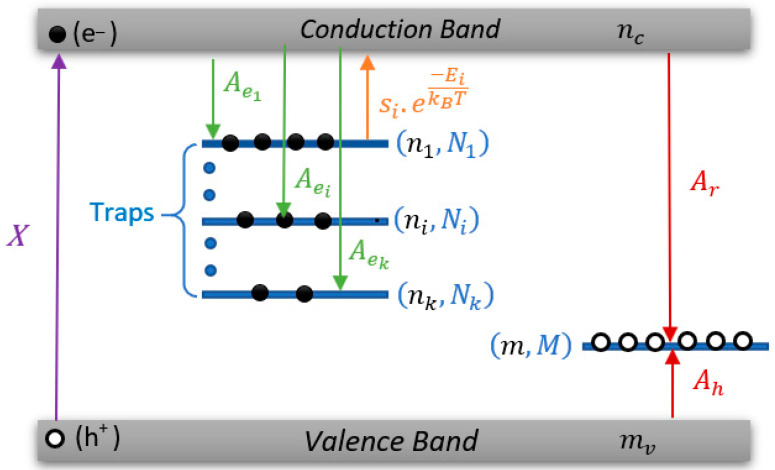
Schematic representation of the levels and processes used in the radioluminescence modeling with *k* electron traps and one recombination center.

The schematic details of the model describing the trapping-detrapping-recombination processes in the active matter are shown in Figure 7. Under X-rays, after the production of electron-hole pairs, electrons have two possible evolution paths. They can be trapped by a set of discrete trapping levels. Then, they may remain trapped or be released, depending on the measurement temperature conditions. The second possibility is to recombine directly with holes on RC, without trapping, by emitting photons. It should be noted that thermal detrapping is not negligible in the process of RL. Hence, the mathematical term describing the electron’s release rate by thermal effect must be considered in the equations.

The simulations proceed by establishing a set of coupled differential equations, which describe the exchange of charges (holes and electrons) between the energy levels, the recombination center, and the conduction and valence bands shown in Figure 7:

(1) $$\frac{dn_{c}}{dt}=X-\sum_{i=1}^{k}n_{c}\left(N_{i}-n_{i}\right){A_{e}}_{i}+\sum_{i=1}^{k}n_{i}s_{i}\exp\left(\frac{-E_{i}}{k_{B}T}\right)-n_{c}A_{r}m$$

(2) $$\frac{dn_{i}}{dt}=n_{c}\left(N_{i}-n_{i}\right){A_{e}}_{i}-n_{i}s_{i}\exp\left(\frac{-E_{i}}{k_{B}T}\right)(i=1,\ldots .,k)$$

(3) $$\frac{dm_{v}}{dt}=X-m_{v}\left(M-m\right)A_{h}$$

(4) $$\frac{dm}{dt}=m_{v}\left(M-m\right)A_{h}-n_{c}A_{r}m$$

(5) $$I_{RL}=n_{c}A_{r}m$$

where the different parameters are defined in Table 1. Equations (1) and (2) represent the variation with time of the electron concentrations in the CB and on the *i*th trap, respectively. Equations (3) and (4) represent the variation with time of the hole concentration in the valence band and recombination center, respectively. Equation (5) describes the RL intensity versus time.

The system of the differential Equations (1)–(5) was numerically solved using the Matlab software package. It should be noted that the number of unknowns is higher than the number of equations. Hence, a program based on the least squares method was developed in order to optimize the transition coefficients (*A_ei_*, *A_r_* and *A_h_*) and reduce the number of unknowns so that it corresponds to the number of equations [16].

Table 2 reports the values of the parameters used in the simulation at different measurement temperatures, including 15 trap levels, which is sufficient to obtain good modeling. Some of these parameters have been obtained from experiments, such as activation energies *E_i_*, frequency factors *s_i_*, the RC concentration *M* and the electron-hole production rate *X*. Other parameters, such as the *A_ei_*/*A_r_* ratio and *N_i_* trap concentrations, have been calculated by fitting the RL signal. It should be noted that the *E_i_* values employed for the cryogenic temperature simulations are taken from reference [18]. More details about the calculation of these parameters may be found in our previous work [16]. These values remain unchanged for all the simulated dose rates from 5 to 150 mGy/s and for all measurement temperatures.

As reported in Table 2, the trap concentrations at cryogenic temperature (from *N*_1_ to *N*_8_) are higher compared to those above RT (from *N*_9_ to *N*_14_), except for the deeper level (*N*_15_), which represents the barycenter of the levels in this energy domain. This latter level is indeed observed in the TL glow curves for all silica matrices doped with rare-earth ions [12,19]. The obtained ratios between transition coefficients *A_ei_/A_r_*, equal to 10^−3^, indicate that the recombination process is predominant over the trapping one, which is in agreement with first-order kinetics and with previous results in silica [20].

**Figure 8:**
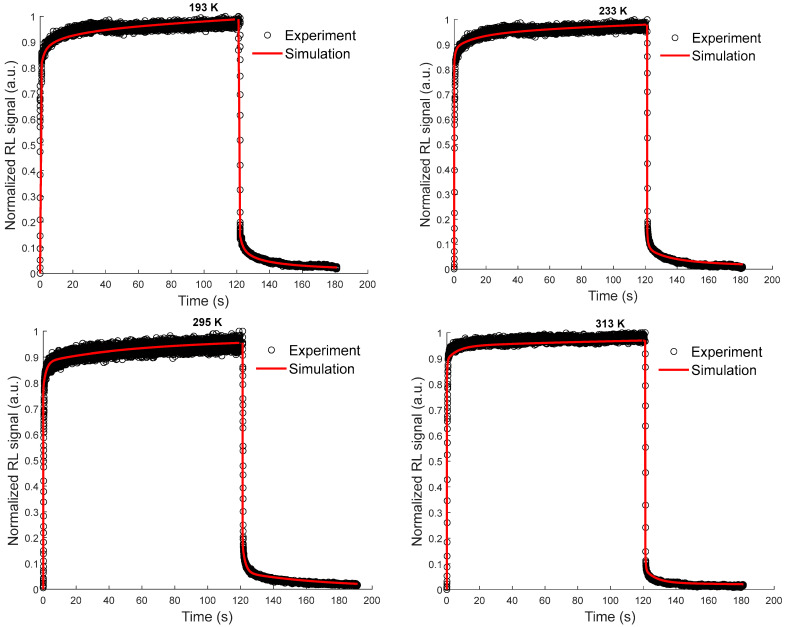

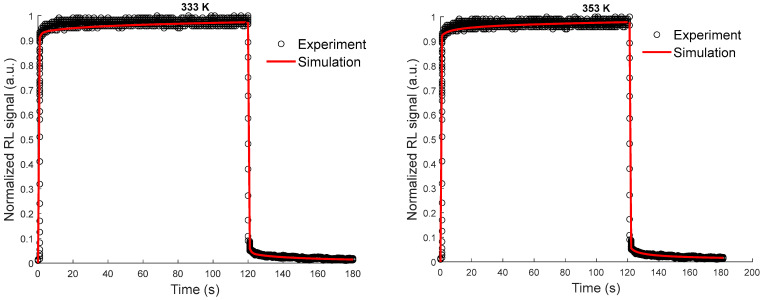
Comparisons of RL signals, measured under X-rays (50 mGy/s) with phosphorescence after the end of irradiation, at different temperature conditions, and simulation results.

Figure 8 presents the results of the simulation as well as those of the experiments for a dose rate of 50 mGy/s at different measurement temperatures, previously shown in Figure 4. As can be observed, the simulations reproduce the RL signals in their different zones at each temperature. It should be emphasized that all the fixed kinetic parameter values used to obtain these simulation results, listed in Table 2, do not change for all dose rates and measurement temperatures. The differences in the RL shape shown at different temperatures in Figure 4 and Figure 8 can be explained considering the trap levels distribution in the matrix bandgap and their concentrations. It depends, in particular, on the metastable trap concentrations at each measurement temperature. For instance, at temperature 193 K, the involved traps (metastable) in the kinetics are the shallowest ones (from *E*_1_ to *E*_3_) and the other traps, given their stability, have less impact on the signal shape. Likewise, at 233 K, the traps at *E*_4_ and *E*_5_ must be added to describe the RL signal shape. Thus, at this temperature, the trap states from *E*_1_ to *E*_5_ are involved. The same logic should be applied to all other temperatures, where deeper traps are activated when the temperature is increased. Hence, the notion of metastable and stable traps is relative to the measurement conditions.

Thereby, the high concentrations of metastable traps at cryogenic temperature (from *N*_1_ to *N*_3,_ as given in Table 2) can explain the slower kinetics and long rise of the signal at the start of the irradiation, as well as the intense and long descent (phosphorescence) after stopping the irradiation. On the contrary, at temperatures higher than RT, the concentrations from *N*_9_ to *N*_14_ being lower, the rise and descent of the RL signal are steeper. It can then be concluded that the higher the metastable traps concentration, the slowest signal rise and descent, and vice versa.

**Figure 9:**
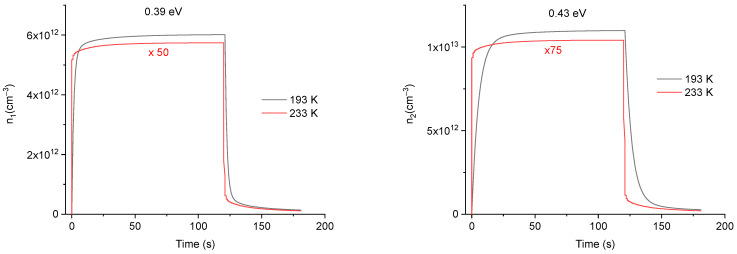

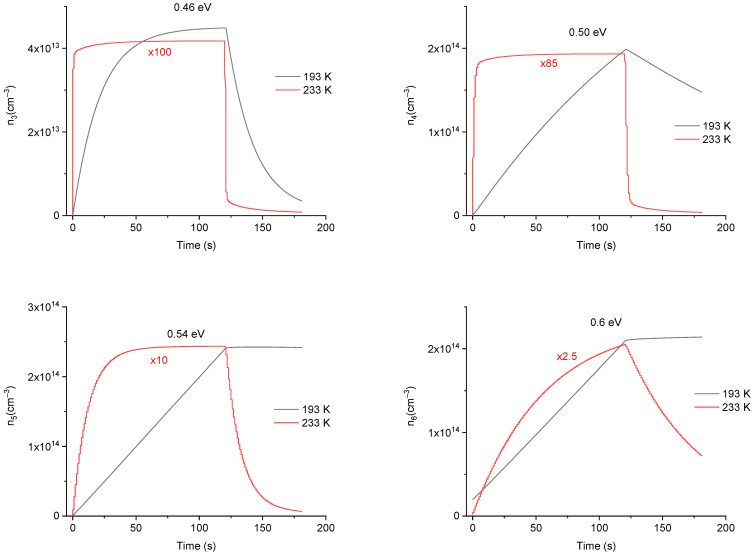
Comparison of the trap charge carriers’ concentrations at different energy levels resulting from the simulation at two measurement temperatures, during and after stopping the irradiation at a dose rate of 50 mGy/s. The multiplying factors applied to the 233 K curves have been chosen to facilitate comparison with temperature 193 K.

Figure 9 shows the simulated time evolution of the charge carriers’ concentrations for different trap levels at temperatures of 193 K and 233 K during and after irradiation. For simplification and legibility, only the electron concentrations of the first six traps have been represented. This Figure 9 clearly shows that the carrier concentration of each trap first begins to increase up to a maximum and then decrease to tend toward an equilibrium state, where $\frac{dn_{i}}{dt}\approx 0$ in Equation (2). However, the dynamics of this common behavior essentially depend on the trap depth. For example, the concentration curves for 0.39, 0.43, and 0.46 eV at 193 K increases quickly during irradiation and show a tendency towards an equilibrium state, where the number of trapped electrons equals the number of released ones. On the other hand, for the trap levels at 0.50, 0.54, and 0.60 eV at the same temperature, their charge concentrations rise slowly without showing any tendency towards an equilibrium state. Similarly, with regard to the phosphorescence part after stopping the irradiation, while the first traps populations (at 0.39, 0.43, and 0.46 eV) show a rapid decrease, the deeper traps populations (at 0.50, 0.54, and 0.60 eV) either show a slight decrease of their population or even remain stable without detrapping. Moreover, the trap populations at 233 K show a rapid trend toward an equilibrium state compared to 193 K, both during and after irradiation. The higher the temperature, the faster the dynamics of the trap populations and the lower the equilibrium concentration.

**Figure 10:**
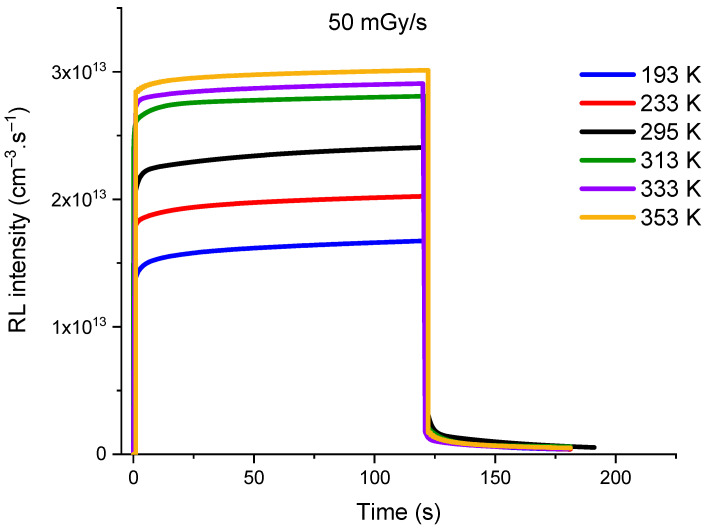
Evolution of the simulated RL signal, for 50 mGy/s X-ray dose rate, at temperature ranging from 193 K to 353 K.

**Figure 11:**
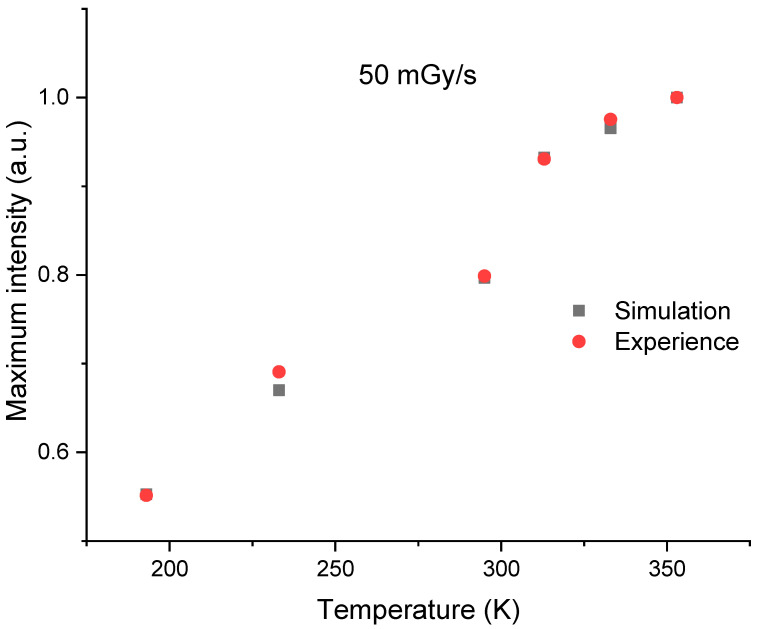
Comparison of experimental and simulated RL signal intensities, after 100 s of irradiation, for a dose rate of 50 mGy/s at different measurement temperatures. These intensities have been normalized to their values at 353 K.

Figure 10 shows the evolution of the simulated RL signal as a function of the temperature for a dose rate of 50 mGy/s. The simulation reflects well the trend of the experimental RL signal evolution presented in Figure 2 for different temperatures from 193 to 353 K. It is important to note that the maximum of the RL signal corresponds to an apparent equilibrium (pseudo-plateau), which in fact is not a real equilibrium. Indeed, the real equilibrium should be reached when the simulated signal intensity *I_RL_* is equal to the rate of electron-hole pairs production *X*, which is 4.3 × 10^13^ cm^−3^.s^−1^ for a dose rate of 50 mGy/s. Figure 11 shows the evolution of experimental and simulated RL signal intensities, after 100 s of irradiation, as a function of the measurement temperature. It should be noted that the experimental and simulated signals, which have been normalized for comparison, present a similar evolution against the measurement temperature.

In order to explain this increase in the RL signal with the measurement temperature, it is relevant to draw on the behavior and the dynamics of the charge trapping/detrapping processes in localized levels, displayed in Figure 9. Indeed, the absolute concentrations of trapped electrons at 0.39, 0.43, 0.46, 0.50, and 0.54 eV are clearly very high at 193 K compared to the ones at 233 K. This indicates that the detrapping increases for these levels when the measurement temperature is raised, which increases the number of electrons available to recombine. As a result, the RL signal intensity increases at higher temperatures. Regarding the trap at 0.60 eV, the concentration becomes of the same order of magnitude for the two temperatures. In effect, as this level lays deeper in the bandgap, the thermal detrapping is much slower and less efficient, even at 233 K. Thus, this trap has a weaker contribution to the RL signal. Finally, it should be stressed that the effect of dose accumulation cannot be negligible both on the RL signal shape and on its evolution as a function of the measurement temperature. On this issue, the effect of the irradiation history of each sample has been considered to define the initial conditions of the charge carrier concentrations in the defect traps and in the recombination center.

## 4. Conclusions

In this study, we present the radioluminescence (RL) response of a sol-gel silica glass rod doped with Ce^3+^ ions under exposure to X-rays at different measurement temperatures. The cerium-doped silica probe presents a broad emission spectrum centered around 460 nm, characteristic of Ce^3+^ ions, for the entire 193–353 K temperature range. The dosimetric characterizations showed that this active material presents an RL signature with a linear behavior as a function of the X-ray dose rate in the 193–353 K range. However, the shape of the RL response with time, namely its dynamical rise toward a pseudo-plateau and its phosphorescence after irradiation, depends on temperature. Furthermore, the overall tendency of the RL intensity is to increase with temperature.

In order to explain this temperature dependence of the RL signal, a model based on several traps and a single recombination center was proposed. The simulation program was based on a standard system of coupled non-linear differential equations, describing the processes of electron-hole pairs generation, trapping-detrapping and recombination. The experimental RL data were compared with those resulting from the simulation to shed light on the effect of the measurement temperature on the RL signal during and after the irradiation run. The RL responses at each measurement temperature were well reproduced by the simulation. The obtained material parameters, such as transition coefficients, agree with previously published works.

In addition, the simulated dynamics of the charge carrier concentrations in the local bands show that when the temperature is increased, the detrapping processes are promoted to the detriment of the trapping ones, explaining the origin of the increase in the signal as a function of temperature. Similarly, the evolution of the trap concentrations is at the origin of the RL signal shape (initial rise, pseudo-plateau and descent). The quite good agreement between simulation and experiment would be helpful to forecast the behavior of such sensors as a function of temperature and should pave the way toward calibration assisted by modeling.

## Figures and Tables

**Table 1:** Description of the model parameters. Fixed parameters have been determined from experimental conditions and from thermoluminescence (TL) measurements. Free parameters have been either adjusted or calculated by the program.

Parameter	Status	Description
*M*	Fixed	Concentration of radiative hole center
$m,\;m_{v}$	Free	Density of holes in *RC* and VB, respectively
*N_k_*	Free	Concentrations of electrons traps (*k* is the number of electron traps)
*n_i_, n_c_*	Free	Density of electrons in *N_i_* and CB, respectively
*X*	Fixed	Rate of electron-hole pairs production
*A_ei_, A_r_*	Free	Electron transition coefficients to traps and RC, respectively
*A_h_*	Free	Hole transition coefficient to RC
*E_i_*	Fixed	Trapping level
*s_i_, k_B_*	Fixed	Frequency factors and Boltzmann constant, respectively

**Table 2:** Values of the used parameters.

Measurement Temperature	Parameter	Value
	*E*_1_ *, E*_2_ *, E*_3_ *, E*_4_ *, E*_5_ *, E*_6_ *, E*_7_ *, E*_8_ *, E*_9_ *, E*_10_ *, E*_11_ *, E*_12_ *, E*_13_ *, E*_14_ *, E*_15_	0.39, 0.43, 0.46, 0.50, 0.55, 0.60, 0.75, 0.85, 0.92, 0.96, 1.01, 1.07, 1.13, 1.18, 1.45 eV
	*N*_1_*, N*_2_*, N*_3_*, N*_4_*, N*_5_*, N*_6_, *N*_7_*, N*_8_*, N*_9_*, N*_10_*, N*_11_*, N*_12_, *N*_13_*, N*_14_*, N*_15_	2 × 10^19^, 1 × 10^19^, 1 × 10^19^, 1 × 10^19^, 9 × 10^18^, 7 × 10^18^, 2 × 10^19^, 1 × 10^19^, 2 × 10^18^, 3 × 10^18^, 3 × 10^18^, 3 × 10^18^, 3 × 10^18^, 3 × 10^18^, 5 × 10^19^ cm^−3^
At all temperature	*s*_1_*, s*_2_*, s*_3_*, s*_4_ , *s*_5_ *, s*_6_ *, s*_7_ *, s*_8_ , *s*_9_ *, s*_10_ *, s*_11_ *, s*_12_ , *s*_13_ *, s*_14_ *, s*_15_	1.16 × 10^10^, 3.51 × 10^10^, 5.2 × 10^10^, 6.59 × 10^10^, 7.2 × 10^10^, 1.76 × 10^11^, 3.94 × 10^12^, 7.54 × 10^12^, 8.94 × 10^12^, 9.88 × 10^12^, 1.51 × 10^13^, 2.11 × 10^13^, 3.45 × 10^13^, 4.03 × 10^13^, 6.66 × 10^13^ s^−1^
	$\frac{A_{e1}}{A_{r}},\frac{A_{e2}}{A_{r}},\frac{A_{e3}}{A_{r}},\frac{A_{e4}}{A_{r}},\frac{A_{e5}}{A_{r}}\;\frac{A_{e6}}{A_{r}},\frac{A_{e7}}{A_{r}},\frac{A_{e8}}{A_{r}},\frac{A_{e9}}{A_{r}},\frac{A_{e10}}{A_{r}}\;\frac{A_{e11}}{A_{r}},\frac{A_{e12}}{A_{r}},\frac{A_{e13}}{A_{r}},\frac{A_{e14}}{A_{r}},\frac{A_{e15}}{A_{r}}$	~10^−3^
	*X*	50 mGy(SiO_2_)/s→ 4.3 × 10^13^ cm^−3^ .s^−1^ 100 mGy(SiO_2_)/s→ 8.6 × 10^13^ cm^−3^ .s^−1^ 150 mGy(SiO_2_)/s→ 1.3 × 10^14^ cm^−3^ .s^−1^
	*M*	2 × 10^18^ cm^−3^
	*t_i_* (Irradiation time)	120 s
	*t_f_* (Afterglow time)	60 s

## Data Availability

The data presented in this study are available on request from the corresponding author. The data are not publicly available due to privacy restrictions.
